# Supplementary material for: Are carbon emissions trading and green financial instruments synergistic? -Comprehensive quantitative research based on content analysis
Source: PLoS One. 2024 Mar 7;19(3):e0298601. doi: 10.1371/journal.pone.0298601 (PMC10919601; doi:10.1371/journal.pone.0298601)
Supplement: S1 Data — (ZIP) [file pone.0298601.s001.zip › Data/地级市Z_F工作报告.zip!/╣π╬≈/╖└│╟╕█╩╨/└┤╫╘╣1⁄2╓┌║┼í╛┬φ┐╦╩2╛▌═°í┐_2012.docx]

各位代表：

现在，我代表市人民政府向大会作工作报告，请予审议，并请市政协委员和列席会议的同志提出意见。

2011年工作回顾

2011年是“十二五”的开局之年。在自治区党委、政府和市委的正确领导下，我市全面贯彻落实科学发展观，进一步转变发展方式，深入实施广西北部湾经济区发展规划，加快启动东兴国家重点开发开放试验区建设，团结拼搏，开拓奋进，克服资金紧张、电力不足、物价上涨等诸多困难，全市经济社会保持持续快速发展的良好势头，较好地完成了市四届人大七次会议确定的年度目标任务。

一、多项指标排全区前列，实现“十二五”良好开局

初步预计，全市生产总值突破400亿元，达418亿元，增长16%，高于全区3个百分点；财政收入突破40亿元，达44.35亿元，增长26.3%；全社会固定资产投资490亿元，增长31.5%。多项指标总量或增幅排全区前列，其中：港口货物吞吐量9024万吨，海关税收超过120亿元，农民人均纯收入6500元，工业经济效益综合指数达590%，均排全区首位；外贸进出口总额、边贸成交额分别达40.5亿美元、24.5亿美元，排全区第二；生产总值、财政收入增幅排全区第三。据中国社科院2011年发布的《中国城市竞争力蓝皮书》，我市综合增长竞争力在全国294个地级以上城市中排名第七。

二、项目建设全面提速，重大基础设施不断完善

实施“项目建设提速年”，狠抓产业百项工程和重点基础设施建设，152个项目共完成投资173亿元，同比增长30%。三大项目加快推进，核电项目一期全面建设，完成投资50亿元，以自主创新技术创建国际安全施工标杆；铜镍项目一期完成投资20亿元，40万吨铜冶炼项目通过环评，铜冶炼主厂房全面施工；钢铁项目完成投资8.7亿元，建成职工休疗养中心，厂区道路、专用码头等配套工程加快推进。钦崇高速公路上思段进入路面施工阶段，南防高铁防城港段基本完成路基工程，防城至东兴高速公路、玉罗岭至李子潭一级公路、贵台至防城二级公路等一批重大交通基础设施全线施工。

三、试验区建设扎实推进，开放合作深入拓展

实施“开放突破年”，开放合作开创新局面。着力推进东兴试验区建设，试验区管委会筹备组挂牌成立并入驻京岛办公；市主要领导多次率队进京赴邕专题汇报，争取国家部委和自治区的支持；试验区实施方案已上报国家发改委，发展规划、体制机制和政策创新研究课题通过专家评审；先行先试，扎实推进三大启动项目，北仑河国际商贸城动工建设，东兴—芒街跨境经济合作区已确定重点建设项目，金滩国际旅游岛项目前期工作加快。东兴口岸改造二期工程快速推进，防城港、江山、企沙等口岸联检大楼加快建设。对外交流与合作成效显著，与越南下龙市签订友好合作备忘录，与意大利热那亚市、波兰热舒夫市签署缔结国际友城意向书，成功举办防城港市首届国际文化交流活动；参加中国—东盟博览会、中国—东盟物流合作论坛等会展活动，签约项目投资额创历史新高；在东盟各国设立办事机构和投资办厂企业数量居全区第一；全市引进区外境内资金266.4亿元，增长115%，实际利用外资1.95亿美元，增长30.8%。

四、城市建设步伐加快，海湾城市特色凸显

继续以“四沿线、四片区、两景观、两改造”为重点，实施城建百项工程361项，完成投资261亿元，城市功能逐步完善，全海景生态海湾城市初具规模。全面推进城市路网建设，城市中心区、桃花湾北部片区、城南新区路网工程及北部湾大道、西湾环海大道、东湾大道等城市干道建设加快。西湾海域环境整治一期工程加快推进。桃花湾体育馆、桃花湖公园一期、市青少年活动中心竣工使用，白鹭公园基本建成。市博物馆、科技图书馆、文化艺术中心完成主体工程。北部湾财经中心、钦防高速防城港收费站及管理区改建等项目开工建设。港口汽车站、防城汽车站顺利搬迁，腾飞广场、北部湾商业中心等旧城改造项目加快推进。一批建筑面积20万平方米以上的楼盘开工建设，房地产开发投资和商品房销售面积总量均排全区第五，增幅分别位居全区第二、第一。实施城乡风貌改造和村镇规划集中行动，编制完成8个乡镇总体规划方案和197个村庄规划。

五、园区经济提速发展，优势产业加快集聚

加快园区基础设施建设，企沙工业区自来水厂、220千伏中路变电站和潭松变电站，大西南临港工业园榕木江大道、污水处理厂等项目加快推进。加快打造千百亿元产业园区，园区集聚效应明显，六大园区规上工业产值达530亿元，占全市84%。大西南临港工业园入园项目91个，园区产值突破190亿元，盛隆镍合金一期项目加快建设，新磷化产业园初具规模。东湾物流加工产业园发展步伐加快，枫叶粮油及大海粮油、嘉里粮油、惠禹饲料蛋白增资扩建等项目建成投产，园区产值突破210亿元。积极扶持企业做大做强，产值超亿元企业达93家，比上年增加16家，总数居沿海三市之首，其中：大海粮油产值突破100亿元，盛隆冶金产值突破80亿元，惠禹饲料蛋白产值突破50亿元。华润水泥二期、桂人堂金花茶精深加工等26项重点技改项目竣工投产，全市完成更新改造投资73.6亿元，增长39.2%。制糖、林产林化、海产品等资源型加工业加快发展。

六、文化旅游蓬勃发展，第三产业快速提升

实施“文化旅游旺市”战略，推动文化、旅游、商贸等产业融合发展。完成旅游基础设施建设投资25.3亿元，白浪滩景区道路和接待服务设施建成使用，南山景区旅游公路建成通车，红沙群岛、皇袍山森林乐园等景区基础设施逐步完善。德城国际大酒店、北港皇冠大酒店等五星级酒店加快建设。新推出红沙群岛海岛游、穿越十万大山边海游等精品线路。屏峰雨林公园创4A级景区工作通过自治区初评，西湾旅游景区、北仑河源头景区被评为3A级景区，东兴古榕度假村等16家村点被评为星级农家乐。旅游城建大篷车开进东北、华北、西北宣传推介，组织全国百家旅行社走进防城港，全年接待国内外游客685.8万人次，增长23%。成功举办京族哈节等“四大节庆”，加快建设《梦幻北部湾》海上实景演出场馆。京族独弦琴艺术被列入第三批国家级非物质文化遗产名录，那良古街保护开发工程开工建设。开展“知行合一”全民读书活动，21项文艺创作获广西“铜鼓奖”等奖项。开展“激情防城港”广场活动，组织各种文艺演出400多场。富裕路商业步行街及北部湾家具广场、月亮岛购物中心等大型商场建成开放，桃花湾集贸市场、品牌汽车冲仑4S店等专业市场开工建设，北京华联、南城百货等大型连锁超市入驻，批零住餐业快速发展，社会消费品零售总额达59.8亿元，增长18.1%；金融、通信、中介等现代服务业加快发展，预计第三产业实现增加值137亿元，增长13.8%。

七、枢纽大港实力增强，现代物流发展壮大

实施“以港立市”战略，加快建设现代化枢纽大港。加强港航基础设施建设，完成投资20.4亿元，18～22号泊位投入试运营，403～407号泊位完成码头水工工程，20万吨级进港航道通过交工验收，云约江南港区前期工作加快推进。成功拓展国际煤炭中转业务，加快打造华南煤炭储备配送中心。成功开通广西首条直航东非的集装箱航线，开辟汽车整车集装箱出口业务。防城港完成货物吞吐量6168万吨，中小港完成货物吞吐量2856万吨。新增航运企业6家、船舶48艘、运力14.2万吨。东湾物流加工产业园路网、防城港东大门、市级粮食储备库、北部湾大货车物流服务中心建成使用，中外运、泰港、汇通等仓储物流基地项目加快建设。冲仑物流园建材、五金机电项目加快推进，茅岭矿业物流园启动建设。

八、全民创业活力迸发，民营经济日益繁荣

实施“全民创业年”，开展就业培训9200人次，实现农村劳动力转移就业2.1万人，城镇新增就业2.3万人，城镇登记失业率2.03%。积极破解创业资金瓶颈，发展小额贷款公司14家，实施青年和妇女创业小额贷款项目，发放创业贷款达7900多万元。新增私营企业1158户、注册资金12.7亿元，均比上年增长一倍以上；新发展个体工商户1万多户。开展全市十强私营企业和十强个体户评选表彰活动。出台扶持政策，实施微型企业发展试点，新注册微企200户。民间投资242.5亿元，增长36.6%，占全市全社会固定资产投资的49.5%，成为投资增长的主要力量。

九、三农基础不断夯实，县域经济快速发展

三农工作取得新成效。农业总产值90亿元，增长5%。粮食实现增产，甘蔗套种技术在全区推广。糖料蔗种植面积达68.8万亩，“红金系列”特色种植达145万亩，水产健康养殖基地达15万亩。防城金花茶被评为国家地理标志保护产品，华石镇火龙果基地列入全国第七批农业标准化示范区。农业产业化重点龙头企业达13家，各类农民专业合作社增至136个。投资4.5亿元完善农村水利设施、村屯道路、安全饮水等基础设施，硬化农村道路220公里。完成集体林权改革。新农村示范点加快建设，“六统强基工程”、“名镇名村工程”深入实施。休闲农业发展形成新趋势，簕山等7个村被列为农业部休闲农业示范点，竹山村被评为广西休闲农业“十佳”名村，“红姑娘”红薯节入选广西十佳休闲农业名节。

县域经济实力明显提升。县域生产总值达275亿元，占全市生产总值的66%。上思县资源型产业发展取得新突破，生态旅游业发展势头良好，获评“中国氧都”，基础设施建设力度加大，全社会固定资产投资、税收增速均超过37%。东兴市全力推进试验区建设，狠抓边贸和旅游业，成功举办第五届中国边境口岸城市市长论坛等重大活动，口岸经济繁荣发展，规上工业产值增长65%以上。港口区全力服务三大项目等重大工程，农民安置区加快建设，公共配套加快完善，工业园区发展壮大，经济实力不断增强，财政收入增长28％以上，规上工业利润增长超过5倍，商品房销售面积占全市比重接近七成。防城区主动服务城建、旅游等重大项目，加大征地拆迁力度，加快旧城改造和新区开发，城市面貌明显改观；大力发展特色农业和商贸、物流业，繁荣城市经济，社会消费品零售总额占全市总量超过40%。

十、民生福祉继续改善，社会事业全面发展

民生工作成效显著。实施“民生改善年”，投资19.3亿元，推进民生项目168个。在全区率先实现新型农村社会养老保险、城镇居民养老保险“全覆盖”，新农合参合率、五保供养标准均居全区前列。在全区率先实施农村义务教育阶段寄宿制学校营养改善工作。加强物价调控，实施价格临时干预，平价供应粮油等生活必需品；建立社会救助和保障标准与物价上涨挂钩机制，市本级向低收入群体发放价格补贴445万元，保障了群众的正常生活。为民办了一批实事，新建6个“菜篮子”基地；新建212个村级卫生室，实现村级卫生室标准化建设全覆盖；建成74个村级公共服务中心、93个农家书屋；开工建设保障性住房7247套，开工率100%；改造农村危旧房3677户，其中农村贫困残疾人危旧房750户；建成市流浪未成年人救助保护中心、儿童福利院和29个五保村；开工建设市老干部交流活动中心、市精神病医院；建成5个垃圾中转站和配套公共厕所；新增公共停车位4950个。

社会事业全面进步。实施“教育振兴工程”，投入2.6亿元，推进教育惠民项目35个；开工建设市委党校新校区和4所乡镇中心幼儿园，建成桃源路小学、北港小学，金海湾小学完成主体工程，成立市第三中学；职教攻坚顺利通过自治区验收，市职教中心完成主体工程。深化医药卫生体制改革，市中医医院成为我市首家“三甲”医院。实施防治艾滋病攻坚工程、母婴健康“一免二补”幸福工程和地中海贫血防治计划。计生优质服务“国优”覆盖率达75%，人口出生率14.33‰，低于责任指标1.67个千分点。参加广西第十二届运动会获金、银、铜牌各13枚，创历史最好成绩。成立自治区级磷化工工程技术研究中心、盛隆新材料研究中心和广西首家红木家具专业鉴定检验室。3—20公里兴边富民行动基础设施建设大会战任务全面完成，第三批整村推进贫困村扶贫开发通过自治区验收。十万山华侨林场体制改革取得突破性进展。全面推进生态文明示范区建设，全市生态环境质量保持良好，获评“中国白鹭之乡”。“五五”普法、第六次全国人口普查均获全区先进。口岸联检、国资管理、气象服务、宗教、档案、修志、妇女儿童、残疾人、红十字、防震减灾等各项社会事业取得新成效。

社会管理进一步加强。依法做好信访工作，积极化解社会矛盾纠纷。市公安局新指挥中心建成投入使用，配备全区最先进的视频指挥系统。警务机制改革取得初步成效，信息化打防控管体制不断完善，社会治安稳控能力和水平不断提高。加强流动人口管理。强化食品药品监督管理。狠抓重点行业和领域安全专项整治，安全生产持续稳定。建立军警民联防联控联管联建机制，加强边海防管理，严厉打击走私等违法犯罪活动。加强国防教育、国防动员体系和民兵预备役建设，扎实推进双拥创建工作，第五次荣获自治区“双拥模范城”。全市社会和谐稳定，民族团结，边疆安宁，人民安居乐业。

十一、政府建设力度加大，发展环境进一步优化

政府执行力不断强化。完成各级政府集中换届工作。编制实施“十二五”规划和各专项规划。进一步理顺市与城区城市规划建设管理、学校医院管理体制和工业园区责权关系。行政审批更加高效，实际办结时间比承诺时间提速11.4个百分点。每月组织一次全市项目集中联合审批现场办公会，加快了项目推进速度。30%的乡镇整合“七站八所”，挂牌建立政务服务中心，市县乡村四级政务服务体系加快构建，政务服务、政务公开和政府信息公开示范点建设卓有成效。

理财水平明显提高。强化税收征管，优化财政支出结构，全市一般预算收入28.3亿元，超额完成年初预算任务，增长24.6%；一般预算支出61.2亿元，增长16.5%。市本级重点向教育、社会保障和就业、医疗卫生、城乡社区、农林水等民生社会事业倾斜，增长均超过60%。项目融资到位资金8.4亿元。对1108个项目进行投资评审，核减不合理资金4.99亿元；投资审计节减资金6100万元；政府采购节约资金1.01亿元，做到少花钱多办事。偿还政府性债务15.25亿元，追回债权资金1亿元，提高了政府信用度。

发展瓶颈进一步破解。积极化解生产要素制约。在用电淡季鼓励企业开足马力生产，在用电高峰积极争取用电指标，科学调度，有效缓解了用电紧张局面。做好建设用地报批工作，获得用地用海2542公顷，是过去五年总和的60%。秉承“文明征地、和谐搬迁”理念，完善征地搬迁补偿安置办法，提高补偿标准，加快安置区建设，征收土地2.6万亩。实施土地整理项目18个，新增耕地579.35公顷，保证全市两年的占补平衡。清理闲置土地8300多亩，集中力量处理好一系列历史遗留问题。

依法行政取得新进展。坚持科学民主决策，强化行政监督和问责。自觉接受市人大的法律监督和市政协的民主监督，广泛听取社会各界意见，办理人大代表建议64件、政协委员提案368件，答复率均为100%。落实廉政建设责任制，深入开展纠风专项治理，严厉查处违法违纪案件，努力营造风清气正的良好环境。

各位代表！

过去一年，在国内外环境复杂多变和面临诸多困难的情况下，我市经济社会发展取得这样的成就，确实来之不易。这是自治区党委、政府和市委正确领导的结果，是市人大、市政协有效监督、大力支持的结果，是中直、区直单位和驻军驻警支持帮助的结果，是各级各部门和全市人民团结拼搏、真抓实干的结果。在此，我谨代表市人民政府，向全市广大干部群众，向解放军指战员、武警官兵和公安民警，向各民主党派、工商联、无党派人士、人民团体，向所有关心、支持、参与我市现代化建设的各界人士和海内外朋友，表示衷心的感谢和崇高的敬意！

在充分肯定成绩的同时，我们也清醒地看到，全市经济社会发展中存在一些不容忽视的问题，主要是：钢铁基地项目尚未大规模建设，缺乏重大产业项目投产，经济增速放缓；电力、用地和节能减排等刚性约束越来越大；城市功能不完善，第三产业发展滞后；高层次高技能人才紧缺，普通劳动力不足的问题开始显现；个别部门和工作人员服务意识、创新意识不强，办事效率不高等。针对这些困难和问题，我们将采取有效措施认真加以解决。

2012年工作安排

根据市第五次党代会精神和市委的统一部署，今年市政府工作的总体要求是：以邓小平理论和“三个代表”重要思想为指导，深入贯彻落实科学发展观，加快转变经济发展方式，坚持以港立市、以开放兴市、以工贸强市、以文化旅游旺市，按照“两步走”的部署，以“定格局、干大事、掀高潮、全面上”为主基调，围绕迎接建市20周年“干成20件大事，大干20件大事”的目标任务，更加突出东兴试验区建设，更加突出打造千百亿元产业，更加突出海湾城市建设，更加突出现代服务业发展，更加突出保障和改善民生，更加突出社会管理创新，奋力跨越追赶，努力争取在广西速度领先、人均领先、质量领先，以优异成绩迎接党的十八大胜利召开！

今年我市经济社会发展的主要预期目标为：地区生产总值增长15%，财政收入增长18%，全部工业总产值增长32%，全社会固定资产投资增长20%，外贸进出口总额增长15%，实际利用外资增长15%，港口货物吞吐量增长10%左右，社会消费品零售总额增长15.5%，城镇居民人均可支配收入增长12%，农民人均纯收入增长13%，居民消费价格涨幅控制在5.5%以下；城镇登记失业率控制在4%以内；人口自然增长率控制在10.5‰以内；万元地区生产总值能耗和主要污染物排放量控制在自治区要求范围内。

要实现上述目标，必须在工作中牢牢把握“定格局、干大事、掀高潮、全面上”的主基调。这个主基调是按照中央和自治区的总基调，结合我市实际提出的。我市作为北部湾优先发展地区，作为国家重点开发开放试验区，作为志在赶超沿海先进地区的新兴城市，必须承担起落实国家发展战略的使命和重任，既要做到“稳中求进”，更要做到“稳中求好、稳中求快、好中求快、快中更快”，抢抓机遇，大干快上，为全区发展多作贡献。

“定格局”就是要把握好北部湾经济区优先发展、同城化进程加快，今年金川铜镍项目将全面施工，核电项目将全面推进，钢铁项目有望获批，东兴试验区实施方案即将获批实施等新形势、新机遇，以更好的作风、更高的效能、更大的力度，推动我市产业发展、城市建设和开放合作新跨越，基本奠定今后10年乃至更长时期的发展格局。

“干大事”就是要在今明两年干成20件大事，大干20件大事，向建市20周年献礼。这两个20件大事，包括基础设施、产业发展、城市面貌、民生福祉、试验区建设等方面的160个项目，关系全局、影响长远，是今明两年政府工作的主抓手，是必须凝聚全市力量共同完成的重大任务。干好这两个20件大事，将实现试验区三大标志性项目全面启动、千百亿元产业集群加快崛起、全海景生态海湾城市基本成型、城乡居民收入持续增长，为实现“两步走”目标奠定坚实基础。

“掀高潮”就是要紧紧抓住一系列重大历史机遇，充分释放近年来我们狠抓基础、大上项目所积蓄的能量，进一步解放思想，大胆先行先试，创优势，打品牌，掀起国际大通道建设新高潮、产业发展新高潮、城市建设新高潮、开放合作新高潮和全民创业新高潮。

“全面上”就是要在重大项目和试验区的带动下，充分调动各方面资源，促进一二三产业全面上、综合实力全面上，努力实现主要经济指标保持速度、人均、质量三个全区领先。到2013年，实现“两个翻番”，即全市生产总值、财政收入比2010年翻一番，工业产值超过千亿元，确保“十二五”目标任务全面完成。

为此，今年要重点抓好以下工作：

一、更加突出东兴试验区建设，加快打造重要门户城市

要大干并尽快干成东兴试验区边贸中心、东兴—芒街跨境合作区和珍珠湾国际旅游区三件大事，努力打造中国—东盟合作先行区和“西部深圳、边境特区”品牌。

全面掀起试验区建设热潮。全力争取国家和自治区的支持，争取试验区实施方案尽快获批。高水平、高标准编制和完善城市总体规划和试验区重点区域控制性详规。东兴试验区边贸中心要加快北仑河国际商贸城、东兴边民互市贸易区二期及国际旅游集散中心建设；东兴—芒街跨境合作区要开工建设口岸综合服务区、拆迁安置区、中央大道等“四区五路”项目；珍珠湾国际旅游区要启动规划，加快金滩国际旅游岛基础设施建设，推进京岛复岛工程实施。加快完成东盟国际中草药产业园、东盟国际会议中心、金沙海景五星级酒店等项目前期工作，力争年内开工。

努力改善通关环境。完善企沙、江山等边地贸口岸规划，积极争取扩大企沙口岸开放范围。加快推进东兴口岸改造工程，建成使用防城港口岸联检大楼，推动广西电子口岸海运物流服务平台二期工程建设，提高联检部门服务质量水平，打造广西通关效率最高、服务最优的口岸城市。

深化与拓展对外开放。优化调整外贸结构，进一步扩大出口，大力发展加工贸易，规划建设红木等进口资源加工区，提升外贸发展水平。做大做强互市贸易，积极发展小额贸易。加强与欧美、东盟各国尤其是越南的合作，充分利用中国-东盟博览会、中越（东兴—芒街）商贸旅游博览会等平台，积极推动文化、旅游、农业、劳务等领域对外交流与合作。积极“走出去”，鼓励引导更多有实力的企业到东盟投资办厂，带动技术、劳务输出。以形成产业集群为目标，按产值大、利税高、能耗低、用地少的要求选商引资、以企引企、招强引大，实现招商引资新突破。

二、更加突出打造千百亿元产业，加快建成新兴港口工业城市

要大干并尽快干成金川铜镍一期二期、红沙核电、防城港钢铁基地和千亿元企沙工业区、东湾物流加工产业园、大西南临港工业园等七件大事，壮大实体经济，打造新兴港口工业城市品牌。

打造千百亿元产业园区。加快完善工业园区供水、供电、路网、排污等基础设施配套，培育优势产业，提升园区发展水平。企沙工业区要全面加快三大项目建设，全年完成投资140亿元以上。金川铜镍项目一期工程全面建设，确保2013年初建成投产；红沙核电项目加快施工进度，尽快开展二期前期工作；钢铁基地项目加快厂区“三通”及专用码头建设，争取获批全面动工。开工建设防城港电厂二期工程等能源项目，大力引进一批钢铁、能源、有色金属等上下游配套产业，加快产业集聚。大西南临港工业园要抓紧清理闲置土地，加快渔洲坪磷化企业搬迁步伐，发展壮大磷硫化工产业，推进盛隆、金源镍业及印刷产业园项目，年内园区产值达到250亿元，力争2013年产值突破500亿元。东湾物流加工产业园要强化园区管理服务，完善园区产业规划布局；惠禹饲料蛋白二期要建成投产，加快大海粮油技改项目建设，做好枫叶粮油二期、天宝重交沥青项目前期工作并早日开工，力争园区产值突破250亿元。

实施“抓大壮小扶微”工程。强化企业服务，完善扶持政策，引导推动企业发展壮大。一是“抓大”。实施“百亿元工业企业工程”和“十亿元工业企业工程”，加快培育盛隆冶金、惠禹蛋白、金川铜镍等企业跨入百亿元企业行列，红沙核电、枫叶粮油、中一重工、中冶实久等企业实现主营业务收入超过10亿元。年内新增百亿元工业企业1家、十亿元工业企业3家、亿元工业企业10家。二是“壮小”。实施“小企业上规模工程”，积极引导、推动一批主营业务收入2000万元以下、有潜质的工业企业壮大规模，年内新增2000万元以上规模企业25家。三是“扶微”。实施“微型企业发展工程”，加大对微型企业的扶持培育力度，年内新增微型企业400家以上。

三、更加突出海湾城市建设，着力完善功能彰显特色

要大干并尽快干成城市中心区、“七大场馆”、西湾景观一期工程、城市综合交通枢纽、广西园林城市、防城江两岸景观开发等九件大事，强化规划引领，完善城市功能，聚集城市人气，不断提升海湾城市魅力。

完善城市规划布局。按照东兴试验区的功能定位，顺应北部湾经济区同城化趋势，坚持以国际眼光、开放视野修编城市总体规划、重点区域控制性详规及各专项规划，对沿海沿边一带进行统一规划布局。

加快城市组团建设。突出片区功能配套，高标准高水平建设，做到建一片成一片。城市中心区要加快“三横两纵”路网、江山大道西段建设，全面建成“七大场馆”并投入使用，加快中央商务区建设，建成金海湾商住区，加快市第四中学、中心区幼儿园、综合市场及一批大型房地产项目建设，尽快形成良好生活居住条件。渔氵万组团要加快桃花湾北部片区公共租赁房、东盟外滩、台湾城等项目建设，推进赤港城市综合体开发；推进珠砂港、仙人湾五组等城中村改造和腾飞广场等旧城改造项目；进一步完善片区教育、医疗等公共服务功能。防城组团要加快珍珠大道等进城口景观打造；全面推进城南新区路网建设，开工建设高铁城北站、长途客运站、公交车站、车站广场，打造城市综合交通枢纽，加快春江嘉园、文苑小区等大型房地产项目建设，带动周边区域开发；推进河西新区路网建设，加快新区开发；加快北部湾商业中心、镇夏市民广场等旧城改造项目建设。公车组团要加快建设滨江小区、锦和家园、沙潭江汽贸城和沙港、和平等安置区。企沙组团要不断完善企沙工业区内生活和商业服务配套，加快北港、大龙等被征地农民安置区建设，把企沙工业区建设成为融产业、城市、生态等功能为一体的宜居宜业新城区。西湾组团要加快西湾环海大道、核电生活基地、大环坞商住区及农民安置区建设，规划建设长榄岛企业总部休闲基地。

抓好城市景观建设。西湾核心景观要加快推进海域环境整治、针鱼岭大桥、西湾海堤、伏波文化园、龙孔墩龙马雕塑景区等项目建设，建成西湾城市沙滩、跨海大桥亮化一期工程。加快桃花湖公园二期工程建设和环湖房地产项目开发。加快防城江两岸城市景观建设，开工建设伏波大道、防城江西岸堤防工程，规划建设文昌大桥及配套道路。创建广西园林城市，实施北部湾大道、金花茶大道、迎宾街景观提升工程，规划建设金花茶公园、蜈蚣岭公园。加大城市风貌的研究和改造力度，凸显海湾城市特色。

加快集聚城市人气。坚持城市与产业融合发展，吸纳更多产业工人和各类人才落户。深化户籍管理制度改革，引导农民有序进城，促进农村人口向城镇集聚。引导和支持房地产业健康发展，重点加快20万平方米以上房地产项目建设。完善商贸、文体、教育、医疗等城市功能配套，推进数字化城市建设，不断提升城市管理水平，以优美的城市环境吸引游客、集聚人气。

四、更加突出现代服务业发展，着力繁荣第三产业

要大干并尽快干成《梦幻北部湾》、江山国际旅游港和五星级酒店三件大事，进一步繁荣文化旅游业，不断壮大第三产业，加快打造海洋文化名市和滨海旅游胜地品牌。

着力繁荣文化旅游业。加快西湾、北仑河口、皇袍山、南山等景区景点基础设施建设，着力将白浪滩、屏峰雨林等景区创建为国家4A级景区。推动九龙潭、野人谷、北仑河源头等漂流景区差异化发展。利用好长寿之乡品牌，培育发展长寿休闲养生产业。精心打造穿越十万大山边海游线路。开工建设防城港旅游码头一期工程，全面运营防城港至越南下龙湾海上旅游航线。深入挖掘、开发伏波文化、京族文化等地域特色文化，创作一批文化精品，加快《梦幻北部湾》海上实景演出场馆及一期配套设施建设。抓好那良古镇等古镇古村的保护和开发，提升国际海上龙舟节等“四大节庆”影响力。发展公益性文化事业，加强乡镇文化站、社区文化活动中心、村级公共服务中心建设与管理，抓好文艺创作。实施20户以下已通电自然村“村村通”广播电视工程。大力发展群众广场文化，承办好第四届广西“魅力北部湾”群众文艺活动。完成刘永福故居等一批重要文物保护单位修缮，加强京族哈节等非物质文化遗产保护。

着力繁荣商贸业。积极构建中心城区商业网络，建成桃花湾集贸市场、品牌汽车4S店、东盟水果交易物流园、沙潭江建材装饰市场、企沙海产品市场、桃花湾花卉市场等专业市场，加快建设桂海·东盟新城大型商场、知名品牌商业广场、辉龙美寓购物中心、越秀百货超市等大型商场超市。继续引导国内外品牌批零连锁企业进驻，推动本地超市升级。完善城乡商业零售网点布局，继续推进“万村千乡市场工程”。加强“农超对接”，引导大型连锁超市与农产品生产基地建立长期产销联盟。坚决打击商业欺诈、制假售假行为。经常性开展“美食节”、“观潮节”、“汽车展”等主题促销活动，进一步扩大消费需求。

着力发展现代物流业。引进和培育发展服务水平高、竞争力强的大型现代物流企业集团，建立完善煤炭、矿石、农产品冷链等专业物流体系，加快发展第三方、第四方物流。完善东湾、冲仑、茅岭、公车等物流园规划，加快园区配套基础设施建设，全面推进东湾保税物流中心和中外运、泰港等在建项目建设，力争开工建设北部湾（防城港）现代物流中心、安派达矿产品仓储物流配送中心、新港物流园等物流项目。

着力繁荣其他服务业。建成德城国际大酒店、北港皇冠大酒店等高端酒店，引进打造一批品牌餐饮企业和示范店，提升住宿餐饮档次。推进房地产业健康快速发展。推动信息咨询、船代货代、法律服务、会计等中介服务业发展。拓展文化、娱乐、体育健身、网络通信等新兴服务业。

五、加速建成亿吨大港，努力建设国际大通道

要大干并尽快干成亿吨大港、东湾大道、防东高速公路、南防高速铁路、钦防高速公路改扩建工程等八件大事，努力打造西部现代化亿吨大港和国际大通道品牌。

建成亿吨大港。加快东湾403-407号泊位后方陆域吹填及配套设施建设，尽快投入生产。加快20-22号泊位专业化改造工程等项目建设。开工建设云约江南作业区泊位工程、东湾513-516号泊位、企沙南港区起步码头及西航道一期工程。力争开工建设30万吨级泊位及配套航道。启动企沙东部离岸岛建设。统筹抓好江山、茅岭等中小港口建设。建成第二铁路调车场，加快建设东湾大道，推进集装箱办理站项目，逐步完善港口集疏运体系。增开航线航班，拓展远洋运输业务，大力开拓货源市场。今年确保形成1亿吨吞吐能力，力争港口货物吞吐量达1亿吨。

加强交通基础设施建设。加快防东高速公路、南防高速铁路建设，建成钦崇高速公路上思段。开工建设钦防高速公路改扩建工程防城港段、广西滨海公路防城港段。加快防东高速铁路、上思至企沙一级公路等项目前期工作。力争开工建设中越北仑河二桥。

六、着力强基础促三农，加快城乡统筹发展

要大干并尽快干成农户万元增收工程、水利建设大会战、“六统强基”工程等五件大事，加强农村基础设施建设，推动特色农业上规模创品牌，立足优势壮大县域经济，加快城乡统筹发展。

进一步夯实农村发展基础。继续加强以水利为重点的农业农村基础设施建设，全面开工建设大垌水库、明江水土综合治理工程，完成病险水库除险加固38座，建成灌区节水改造配套加固工程25个、渠道“三面光”160公里、标准海堤工程25公里；新增恢复农田有效灌溉面积8万亩以上。加强乡镇政权基础设施规范化建设，实现全市行政村“六统强基”工程全覆盖。

深入实施农户万元增收工程。稳定粮食生产，大力发展优势特色产业，扩大甘蔗套种、“红金系列”特色产业及无公害标准化蔬菜种植面积，大力发展畜牧水产健康养殖，积极发展林下经济和生态休闲农业。加大农产品加工企业引进、培育和扶持力度，做大做强水产品加工业，在港口、防城、东兴各新规划建设一个适当规模的水产品加工园区，加快推进金鲳鱼等产业化开发项目，打响农产品知名品牌，规模以上农业产业化龙头企业要达到30家以上。加强农村专业合作组织建设，会员50人以上的专业合作社达20个以上。发展劳务经济，加大农民培训力度，提高农民创业技能，实现农村劳动力转移就业2万人以上。加快新农村建设，强化村镇规划，继续抓好和提升红沙、冲敏、竹山、平江等16个生态农业和休闲旅游农业示范点。新建新农村建设示范村12个，建设万元增收工程和林下经济示范点各15个。建成人均纯收入超万元的村屯100个以上，培育户均纯收入超10万元的“示范农户”200户以上。

进一步做特做强县域经济。各县（市、区）要围绕功能定位和发展重点，发展特色经济，增强发展活力。上思县要稳定甘蔗种植面积，加快朗姆酒产业基地建设，延伸制糖产业链，壮大林产林化、水泥建材、生态旅游等特色支柱产业，大力发展林下经济，加快打造北部湾生态后花园和生态经济强县。东兴市要敢于并善于先行先试，推进试验区建设，壮大边境贸易、进出口资源加工、边境旅游等优势产业，大力发展微型企业，加快城区东拓，抓好江平新城规划，努力建设沿边开发开放示范区。港口区要继续服务好重大项目，突出抓好征地搬迁安置，加快大西南临港工业园建设，发展商贸、物流、房地产等现代服务业，完善城市功能，发展城市经济，打造广西北部湾率先发展核心区。防城区要着力打造沿海沿边沿江经济带，重点发展壮大城郊农业和商贸、旅游、物流业，做好重大项目征地搬迁安置工作，加快“一江两岸”等城市新区开发建设，打造产业配套基地、后勤保障基地。

七、积极抓好财政金融，努力破解资金瓶颈

发挥财政资金作用。落实中央积极的财政政策，发挥财政资金的引导、放大作用，全力支持打造千百亿产业，支持工业园区建设，支持企业做大做强，以企业发展促进财政增收。认真落实各项强农惠农政策，继续加大对农业发展支持力度，支持打造特色农业，巩固农业的基础性地位。用足用活财政政策，大力支持商贸、特色旅游、交通运输、现代物流等服务业发展，增强全市经济活力。

拓宽融资渠道。继续搭建好政银企沟通平台，大力引进银行、保险、证券、担保等金融机构，优化信贷投放结构，稳步推进小额贷款公司、融资性担保公司的发展，加大对中小企业、微型企业的信贷支持。成立东兴试验区投资集团。积极培育企业上市。扩大跨境贸易人民币结算试点。整合壮大、规范管理投融资平台，推动BT、BOT等多元化融资。

强化税收征管。发挥综合治税职能，突出抓重点税源、重点企业的税收征管，统筹抓好常规税收征管，确保应收尽收。加强政府非税收入征管，深挖财政增收潜力。要在确保收入及时、足额、均衡入库的基础上，切实提高财政收入质量。

保障重点支出。优化支出结构，加大民生等重点支出，优先保证重点在建、续建项目的资金需求。严格控制一般性支出，厉行节约。加强债务管理，防范财政风险。

八、更加突出保障和改善民生，持续提升人民群众幸福感

要大干并尽快干成城乡居民基本社会保障全覆盖、教育振兴工程五大项目、渔氵万岛生态环境综合整治工程、保障性住房建设等五件大事，以更大的投入改善民生，以强有力的措施保障民生，努力打造广西民富第一品牌，加快建设幸福和谐防城港。

深入推进教育振兴工程。实施学前教育三年行动计划，加快全市中小学布局调整和校舍标准化改造，改善农村学校寄宿条件，桃源路小学、北港小学、金海湾小学要完善建设并对外招生，逐步解决城区义务教育“大班额”问题。加强师资培训，强化教学管理，提高教育质量。加快市职教中心建设，确保广西财经学院防城港校区新生入学。

强化医药卫生服务。扎实推进医改工作，加强公共卫生体系建设，完善农村三级卫生服务网络，发展社区卫生事业。加快市第一人民医院创“三甲”步伐。加快市儿童医院前期工作并尽快开工建设。抓好县级公立医院改革。继续实施防治艾滋病攻坚工程。深入推进诚信计生，大力开展优质服务，加强出生人口性别比综合治理，推进信息人口计生工程建设，全面提升出生人口素质。

提升社会保障水平。推动城乡居民基本社会保障全覆盖，大力实施城乡居民社会养老保险，确保参保率75%以上，实现符合条件的城乡老年人都可以享受养老保险待遇，发放率达100%。巩固提高城镇职工基本医疗保险参保率和新型农村合作医疗参合率。规范城乡低保管理，提高保障标准。认真落实孤儿养育保障、老年人优待等政策，健全弱势困难群体档案，提高社会救助和社会福利水平。继续做好重大项目被征地农民社会保障工作。加强保障性住房的建设、管理和运营。

促进全民奋发创业。加强就业扶持，抓好职业培训体系建设，与企业共建培训基地、创业示范基地、企业孵化基地，重点做好被征地农民、城乡新生劳动力、就业困难人员等重点群体的就业工作，促进经济增长与扩大就业良性互动，年内新增就业2.3万人。优化创业环境，落实政策措施，规范市场行为，大力发展民营经济，进一步激活民间投资。引导群众转变就业创业观念，倡导实业致富，大力提高中等收入者比重。

加强人才科技工作。实施“九大重点人才工程”，加强专业技术人才队伍和高技能人才队伍建设。大力实施“六大科技工程”，落实厅市会商重大科技项目，全力推进优势特色产业的科技创新，规划建设高新技术园区。加大知识产权保护力度。

实施新一轮扶贫开发攻坚战。按照国家新的扶贫标准，把全市25.3万农村贫困人口列入扶贫对象。实施贫困村整村推进扶贫，抓好基础设施建设、重点产业开发、劳动力培训、扶贫到户贴息贷款等工作。深入推进社会扶贫，做好机关及企事业单位定点扶贫和对口支援。深入推进新一轮兴边富民行动，改善边境地区尤其是少数民族聚居点生产生活条件。

更加突出社会管理创新。进一步创新信访工作机制和方法，健全维护群众权益机制，做好社会稳定风险评估。完善城乡社区自治和服务组织建设，健全新型社区管理和服务体制，提高城乡基层组织社会管理服务能力。建立专门队伍，研究解决征地拆迁中存在的历史遗留问题。完善公共安全体系和应急救援体系，强化安全生产监管，遏制伤亡事故特别是较大以上事故发生。完善大巡防机制，推进社区警务和监控建设。推进社会管理信息化建设，加强流动人口服务和网络信息管理。加大扫黄打非力度。深入打击和防范跨境走私、贩毒等犯罪活动，加强社会治安综合管理，不断提高人民群众的安全感和满意度。深入推进十万山华侨林场体制改革。

加强生态环境保护。全面实施《防城港市生态建设规划》，加强节能减排工作，积极发展循环经济和低碳经济，推进生态文明示范区建设。加快城镇污水管网、垃圾处理设施的规划和建设。实施渔氵万岛生态环境综合整治工程，治理渔氵万港区扬尘，建成沙潭江中心区防洪防潮二、三期工程。加强北仑河口、防城江等重要生态功能区、流域的保护与治理。全面推进农村环境连片整治示范建设，积极开展生态文明试点创建。深入实施“绿满八桂”造林绿化工程。

做好“双拥共建”及其他工作。大力推进“双拥共建”活动，巩固和发展军政军民团结。进一步加强边海防管理、国防教育、国防动员、国防后备力量和人民防空工作，维护边疆民族地区安全稳定。充分发挥工会、共青团、妇联、红十字、残联等群团组织的作用。继续做好侨务、宗教、统计、食品药品安全、气象服务、防震减灾、国资管理、档案、修志等工作。

着力为民办实事。在全面落实自治区为民办实事项目的基础上，结合我市实际，集中财力为民办好一批实事：新建、改扩建23个城区、乡镇公办幼儿园；新建36个村级公共服务中心，8个社区服务中心（站）；建设好10个标准化“菜篮子”工程基地；新建4个城区便民菜市场和50个农家便民店；新建1个社区体育馆；新建农村硬化道路200公里；新建25个城市公交候车亭；新开工建设保障性住房5330套，竣工3400套，改造农村旧危房4000户；完成农村和学校饮水安全工程70处，解决3.7万人安全饮水问题。

各位代表！要完成今年的目标任务，必须加强政府自身建设，加快推进政府职能转变和管理创新，重点打造务实、法治、廉洁的服务型政府。深化行政审批制度改革，简化审批流程，完善电子政务平台，提高行政效率。成立重大行政决策咨询委员会，建立健全政府决策咨询机制。加强民主法治建设，自觉接受人大及其常委会的工作监督和政协的民主监督，认真听取各民主党派、工商联、人民团体和社会各界人士的意见。健全政府信息发布制度，推进政务服务和政务公开向基层延伸，提高政府透明度。坚持深入基层、深入实际，体察民情，纾解民困。倡导“马上办”的工作作风，落实和完善领导联系重点项目制度和现场办公制度，及时协调解决实际问题。深入开展“学文件、用政策”活动。全面落实党风廉政建设责任制，健全完善惩治和预防腐败体系，严查腐败案件。加大重大项目的监管力度，继续加强财政监督和政府投资审计，完善招投标机制，建设优质工程、安全工程、阳光工程、廉洁工程。

各位代表！

春潮涌动金龙舞，港城崛起骏马驰。新的一年，任务艰巨而繁重，责任重大而光荣。让我们在市委的坚强领导下，团结和依靠全市各族人民，解放思想，只争朝夕，奋勇争先，跨越追赶，为建设“幸福和谐防城港”而努力奋斗！
